# Autologous Paracrine Prostasin–Matriptase Serine Protease Interaction in Lymphoid Cancer Cells

**DOI:** 10.3390/cells14040247

**Published:** 2025-02-10

**Authors:** Li-Mei Chen, Karl X. Chai

**Affiliations:** Division of Cancer Research, Burnett School of Biomedical Sciences, College of Medicine, University of Central Florida, Orlando, FL 32816, USA

**Keywords:** serine protease, prostasin, matriptase, proteolytic activation cascade, exosome, lymphoma, cluster of differentiation molecules, CD14, CD95

## Abstract

The serine protease prostasin on the surface of the exosomes released from epithelial cells can interact with ectopically over-expressed cell-surface serine protease matriptase in cancerous B cells to initiate the prostasin–matriptase proteolytic activation cascade. Matriptase activation and the ensuing self-activation result in its removal from cancer cells, reducing cell proliferation and migration. In this study, we tested the hypothesis that the matriptase in the lymphoid cells could be removed by the prostasin-initiated activation and self-activation using genetically engineered autologous cells carrying prostasin. In co-cultures with the prostasin-positive cells, the matriptase on the prostasin-negative vector-control cells was removed in a dose-dependent manner, as determined by flow cytometry. This paracrine phenotype requires the active sites of both proteases. In silico analysis of the RNA-seq profiles indicated an imbalanced expression of high matriptase and low prostasin, and their cognate protease inhibitors in B-cell lymphoma patient specimens. The impact of exosomal prostasin on the cluster of differentiation molecules in activated human peripheral blood mononuclear cells was investigated by flow cytometry, revealing candidate mechanisms for prostasin’s role in regulating cellular adaptive immunity. This autologous paracrine prostasin–matriptase interaction could be exploited as a method for targeting over-expressed matriptase in diseases such as B-cell lymphoma.

## 1. Introduction

The diverse functions of serine proteases are intrinsic to their structures and affected by their tissue or cell locations. Most serine proteases are secreted outside the cell after biosynthesis and function as soluble proteins [1]. The extracellular membrane serine proteases, such as matriptase and prostasin, can function in situ in the cell membrane, or in the soluble form after being shed off from the cell membrane into the surrounding environment, or on the surface of the exosomes released from the cell [2,3,4,5]. The shed-off soluble or exosomal membrane surface proteases can reach and affect cells in other tissues or organs. Matriptase is a type-II transmembrane protein, while prostasin is anchored to the membrane by glycosylphosphatidylinositol (GPI) [6,7,8,9]. Matriptase and prostasin are capable of activating each other’s zymogen in a reciprocal fashion. Both enzymes are almost exclusively co-expressed in all epithelial cells. An absence of either protein in the epithelium is detrimental and results in severe dehydration in neonatal skin in mice [10,11,12].

These two extracellular membrane serine proteases perform their physiological functions coordinately, relying on their reciprocal proteolytic activation. Matriptase, however, has an auto-activation mechanism to activate additional zymogens once the initial activation is triggered. Activation and the ensuing auto-activation result in matriptase shedding from the cell surface [13,14,15]. The prostasin–matriptase proteolytic activation cascade in a normal epithelium is tightly regulated by two Kunitz-type transmembrane serine protease inhibitors, hepatocyte growth factor activator inhibitor-1 and -2 (HAI-1 and HAI-2) [16,17,18,19,20]. The prostasin protein on the cell membrane is proteolytically active, while most of the active matriptase molecules are immediately inhibited by HAI-1 on the cell surface or in the extracellular space [9,13,18,19,20,21]. The activity of matriptase is regulated by HAI-2 in a prostasin-dependent pathway [14,22,23,24]. Prostasin is often down-regulated or absent in advanced epithelial cancers. Upon re-expression, prostasin functions as a tumor suppressor, reducing tumor growth, invasion, and the metastatic potential [2,25].

Lymphoma is a cancer of the lymphatic system, including Hodgkin lymphoma and non-Hodgkin lymphoma (NHL). NHL accounts for about 90% of all lymphoma cases and is one of the most common cancers in the US [26]. Lymphoma is also common in children and teens, accounting for about 12% of all childhood cancers. Within the scope of NHL, Burkitt lymphoma is the fastest growing, and involves very aggressive tumors, accounting for 1–2% of NHL cases in all age groups, but, strikingly, 40–50% of all childhood cancers in equatorial Africa [27,28]. Matriptase is ectopically over-expressed in about 50% of B-cell lymphoma specimens, and 86% of Burkitt lymphomas expressed matriptase alone without its cognate inhibitor HAI-1 [29,30,31]. In the absence of HAI-1 co-expression, matriptase becomes highly oncogenic to induce spontaneous cancers and promote carcinogenic tumor formation [32,33]. Reducing the matriptase levels or re-expressing HAI-1 reverts the oncogenic phenotypes associated with the matriptase over-expression. Lower levels of matriptase in cancer cells were associated with delays in tumor formation and growth in vivo in mouse models [34].

Prostasin is found in the exosomes released into bodily fluids or tissue culture media [2,9,35,36,37]. In recent years, prostasin exosomes in the circulation or bodily fluids have been studied as potential diagnostic biomarkers in various diseases or conditions, e.g., in the urine of patients with primary aldosteronism, essential hypertension, or albuminuria [38,39,40,41]; in the blood of patients with severe coronavirus disease-2019 (COVID-19) [42]; and in the saliva of patients with oral squamous cell carcinoma [43]. Importantly, we have demonstrated that the prostasin in the exosomes retains its serine protease activity [36]. Furthermore, we have recently postulated that active prostasin on the exosome surface could activate and shed off matriptase from cancerous B cells upon an exosome-cell contact. This was achieved using several NHL cell lines with an ectopic over-expression of matriptase [37]. The activated matriptase was removed from the cancer cells and as a result, cancer cell growth and migration were reduced in the in vitro cell culture setting. The prostasin exosome activation–elimination of matriptase also induced cancer cell death.

This study aims to explore the potential of adapting prostasin antitumor action targeting the matriptase in B-cell lymphoma to an autologous cell–cell interaction, akin to the mature technology of chimeric antigen receptor T cell (CAR-T) therapy [44,45]. We present data to show that prostasin and matriptase form a proteolytic protease activation cascade in lymphoid cancer cells by an autologous paracrine mechanism. This was achieved by genetically modifying the matriptase-positive B cells to produce prostasin, and co-culturing with the cells harboring an empty vector. We also evaluated the RNA-seq data profiles of the protease–inhibitor network proteins including matriptase, prostasin, HAI-1, and HAI-2 in epithelial versus hematological cancers using web-based tools. We further evaluated the impact of the exosomal prostasin on the cluster of differentiation (CD) molecules in activated human peripheral blood mononuclear cells (PBMCs). This study opens the possibility of exploiting prostasin exosomes or autologous prostasin-enriched cells as a tool in hematological disease interventions.

## 2. Materials and Methods

### 2.1. Cell Culture

The Namalwa (ATCC^®^ CRL-1432™) human Burkitt lymphoma cell line, Jurkat (ATCC^®^ TIB-152™) human acute T cell leukemia cell line, and HEK293T (ATCC^®^ CRL-3216™) human embryonic kidney cells used were purchased from the American Type Culture Collection (ATCC, Manassas, VA, USA). The T-REx™ Jurkat Cell Line was purchased from Invitrogen (part of Thermo Fisher Scientific, Waltham, MA, USA) and was used to construct sublines for over-expressing matriptase variants upon induction with tetracycline. All cells were maintained according to the manufacturers’ instructions in an incubator at 37 °C with a humidified atmosphere of 5% CO_2_ in air. Tissue culture flasks and dishes were purchased from Sarstedt, Inc. (Newton, NC, USA). Heat-inactivated fetal bovine serum (FBS) was purchased from Sigma-Aldrich (St. Louis, MO, USA). Other cell culture media and reagents were purchased from Thermo Fisher Scientific (Waltham, MA, USA).

### 2.2. Establishment of Sublines Over-Expressing Prostasin or Matriptase

HEK293T cells were used to generate the HEK293T-Pro subline to express the wild-type human prostasin and HEK293T-Vec, carrying an empty pLVX-Puro vector (Clontech laboratories, Inc., Mountain View, CA, USA), using lentiviruses and procedures described previously [9]. The Namalwa sublines NamaTR-Pro and NamaTR-ProM for tetracycline-regulated (TR) expression of the wild-type human prostasin or a protease-dead serine active-site mutant, respectively, were constructed and maintained as described previously [37]. The T-REx™ Jurkat sublines JKTR-MT and JKTR-MTM were established for tetracycline-regulated expression of the wild-type human matriptase and a protease-dead serine active-site mutant, respectively. The Jurkat subline JK-Pro was constructed to over-express the wild-type human prostasin. The sublines harboring the appropriate empty lentiviral vectors, NamaTR-Vec, JKTR-Vec, and JK-Vec, respectively, were also generated and used as the controls in the experiments. The expression of the appropriate recombinant protein in the sublines was confirmed by Western blotting as described previously [36,37].

### 2.3. Autologous Cell–Cell Co-Cultures

The cells used for these experiments were labeled with CellTrace Violet or CellTrace CFSE (Thermo Fisher Scientific, Waltham, MA, USA) before addition to the co-cultures. The labeling was performed using the manufacturer’s procedures and described previously [36]. Briefly, the NamaTR-Vec cells were collected and washed with the RPMI medium to remove the FBS in the growth medium. The cells were then incubated with CellTrace Violet at a final concentration of 5 μM for 20–30 min at the room temperature. The excess dye was inactivated by adding the growth medium (with FBS), and the cells were recovered by centrifugation, removing the dye in the supernatant. After labeling, a fraction of the cells was analyzed by flow cytometry to confirm the proper dye incorporation, and the single-colored cells were used for compensation calculations in flow cytometry analysis. The labeled cells were resuspended and cultured in the growth medium (with FBS) overnight before setting the co-cultures. The NamaTR-Pro or NamaTR-ProM cells were subjected to the same procedures above, but labeled with CellTrace CFSE at a final concentration of 2.5 μM. The JKTR-MT or JKTR-MTM cells were labeled with CellTrace Violet and the JK-Pro cells were labeled with CellTrace CFSE. For cell–cell co-cultures, the NamaTR-Vec cells were mixed with the NamaTR-Pro or the NamaTR-ProM cells at a ratio of 1:1, or 1:2, or 1:3 in the Opti-MEM I medium (Gibco, part of Thermo Fisher Scientific, Waltham, MA, USA) and cultured for 24 h in the presence of tetracycline (tet, 1 μg/mL) and tumor necrosis factor-alpha (TNFα, 5 ng/mL) for an enhanced activation of the CMV promoter of the recombinant cDNAs [46]. Altogether, there were 13 samples in the co-culture experiment, including the NamaTR-Vec cells alone, NamaTR-Vec + NamaTR-Pro (1×), NamaTR-Vec + NamaTR-Pro (2×), NamaTR-Vec + NamaTR-Pro (3×), NamaTR-Vec + NamaTR-ProM (1×), NamaTR-Vec + NamaTR-ProM (2×), NamaTR-Vec + NamaTR-ProM (3×), JKTR-MT cells alone, JKTR-MT + JK-Pro (1×), JKTR-MT + JK-Pro (2×), JKTR-MTM alone, JKTR-MTM + JK-Pro (1×), and JKTR-MTM + JK-Pro (2×). The experiment was repeated once in the Opti-MEM I medium. In a separate experiment, each subline was labeled with dyes and treated with tet and TNFα first in the growth medium (with FBS) for overnight before setting the co-cultures for another 24 h. The NamaTR-Vec or JKTR-Vec cells without or with dye labeling were cultured alone for use as the controls in flow cytometry.

### 2.4. Exosome Isolation and Co-Cultures

The HEK293T sublines (Vec and Pro) were cultured to confluence and the conditioned media were collected for exosome (Vexo and Pexo) isolation as described previously [36,37]. Briefly, the conditioned media were centrifuged stepwise at 300× *g* for 5 min, and at 3000× *g* for 15 min to remove cell debris and large vesicles. The supernatant was mixed with polyethylene glycol (PEG8000) at a final concentration of 8.3% for 18–24 h. Exosomes were collected as pellets after centrifugation at 1500× *g* for 30 min. The exosome pellets were suspended in phosphate-buffered saline (PBS, pH 7.4) and re-pelleted by ultracentrifugation at 100,000× *g* for 90 min. This step further purifies the exosomes and removes the soluble proteins carried over from the culture medium. The total protein concentration in the isolated exosomes was determined using the Pierce™ BCA Protein Assay Kit (Thermo Fisher Scientific, Waltham, MA, USA). For the cell–exosome co-cultures, the NamaTR-Vec cells expressing an endogenous matriptase, the JKTR-MT cells expressing a recombinant wild-type matriptase, or the JKTR-MTM cells expressing a recombinant protease-dead mutant matriptase were treated with tet and TNFα for 24 h in a 37 °C incubator before co-culturing with Vexo or Pexo, for a total of 6 samples for the 3 cell types paired with the 2 exosome types. The exosomes were added at a final concentration of 25 µg/mL, and the cell-exosome co-cultures were incubated for another 24 h. Cells co-cultured with PBS were used as the controls for a total of 3 samples for the 3 cell types. The experiment, with a total of 9 co-culture samples for the 3 cell types, was repeated twice.

### 2.5. Cell-Surface Marker Screening

Human peripheral mononuclear cells (PBMCs) were purchased from HumanCells Biosciences (Milpitas, CA, USA) or the ATCC (Manassas, VA, USA). Cells were thawed according to the manufacturers’ instructions and resuspended in the RPMI medium supplemented with 10% heat-inactivated FBS, 1 mM sodium pyruvate, 2 mM L-glutamine, non-essential amino acids (1×), and antibiotic-antimycotic agents (1×). The cells were seeded in U-bottom 96-well plates at 3 × 10^5^ per well in 50 μL of medium containing 2.5 μg of the vector control or the prostasin exosomes (Vexo or Pexo). The cells were incubated at 37 °C for 1 h before the addition of another 50 μL of medium containing lipopolysaccharides (LPS 026:B6, Sigma-Aldrich, St. Louis, MO, USA) and phytohemagglutinin (PHA, Sigma-Aldrich, St. Louis, MO, USA), at 10 μg/mL for each. The cells were then cultured for another 18 h before antibody labeling using the BD Lyoplate™ human cell surface marker screening panel per the manufacturer’s instructions (BD Life Sciences, Franklin Lakes, NJ, USA). Briefly, the cultured cells in the 96-well plates were spun, washed, and blocked with Human BD Fc Block™ (BD Life Sciences, Franklin Lakes, NJ, USA) in 100 μL of BD Pharmingen Stain Buffer/EDTA (flow buffer). Twenty microliters of each antibody solution were added to each sample and the plates were incubated on ice for 20–30 min. The cells were then washed twice and resuspended in the buffer containing the secondary antibody (1:200) and incubated for 20–30 min on ice in the dark. After washing away the secondary antibody, the cells were fixed with 4% paraformaldehyde in PBS for 10 min, washed and resuspended in the flow buffer in the dark and kept at 4 °C for overnight. On the next day, the cells in the 96-well plates were analyzed by flow cytometry using the CytoFLEX S instrument (Beckman Coulter, Brea, CA, USA). The experimental controls were the unstained cells, cells with PBS or exosomes alone, and cells with the secondary antibody alone. BD APC Annexin V or 7-AAD (BD Life Sciences, Franklin Lakes, NJ, USA) was used for differentiating live and dead cells and for gating. The toll-like receptor 4 (TLR4) antibody (BD Life Sciences, Franklin Lakes, NJ, USA) was included in the screening assay. The antibody list is provided in Table S1.

### 2.6. Flow Cytometry Antibodies and Data Analysis

The following antibodies were used: the M32 matriptase monoclonal antibody, 1:200 dilution (a gift from Dr. Chen-Yong Lin, Georgetown University, Washington, DC, USA) [47]; and a goat anti-mouse IgG-Alexa-647™, 1:200 dilution (BD Life Sciences, Franklin Lakes, NJ, USA). The labeled cells (10,000) were analyzed using the CytoFLEX S flow cytometer (Beckman Coulter, Brea, CA, USA). Un-colored and single-colored cells were used for compensation calculations. The flow cytometry data were analyzed using the FlowJo™ v10.9.0 Software (BD Life Sciences, Franklin Lakes, NJ, USA).

### 2.7. Gene Set Expression Analysis

Gene expression profiles of matriptase (gene symbol *ST14*), prostasin (gene symbol *PRSS8*), HAI-1 (gene symbol *SPINT1*), and HAI-2 (gene symbol *SPINT2*) were visualized using publicly available web-based tools at the UCSC Xena Functional Genomics Browser (University of California Santa Cruz, https://xenabrowser.net/, accessed on 20 December 2024) and the Genomic Data Commons (GDC) Data Portal, https://portal.gdc.cancer.gov/, accessed on 24 December 2024. These tools provide mRNA expression (RNA-seq) data for researchers to identify specific molecular changes in cancers. The RNA-seq datasets used in this study are normalized, log-transformed, de-identified, and publicly accessible, and can be downloaded for analysis without ethics review and approval.

A carcinoma dataset of The Cancer Genome Atlas (TCGA) Program PanCan Atlas project was used to compare gene expression across carcinomas of epithelial origin. We built a cohort of 8 common cancer types including BRCA (breast invasive carcinoma), LUSC (lung squamous cell carcinoma), ESCA (esophageal carcinoma), LUAD (lung adenocarcinoma), OV (ovarian serous cystadenocarcinoma), PRAD (prostate adenocarcinoma), STAD (stomach adenocarcinoma), and COAD (colon adenocarcinoma). A cohort of three blood cancer datasets was generated using the Cancer Genome Characterization Initiatives program–Burkitt Lymphoma Genome Sequencing Project (CGCI-BLGSP), the NCI Center for Cancer Research program–Diffuse Large B Cell Lymphomas Project (NCICCR-DLBCL), and the Cancer Genome Atlas program–Acute Myeloid Leukemia Project (TCGA-LAML). The key words of “age at index” and “treatment outcome” were used for stratification of the patient groups. The downloaded datasets were analyzed in Microsoft Excel.

### 2.8. Statistical Analysis

The results from experimental groups in repeat experiments were analyzed in Microsoft Excel and were presented as mean ± standard deviation (SD). Student’s *t* test was used to compare the means between two groups, in which a *p* value less than 0.05 was considered statistically significant. One-way analysis of variance (ANOVA) coupled with the Tukey post hoc test was used to assess the results from three or more independent groups, in which a *p* value less than 0.05 was considered statistically significant.

## 3. Results

### 3.1. Autologous Prostasin–Matriptase Serine Protease Interaction Reduced the Endogenous Matriptase Content in the Namalwa Human Burkitt Lymphoma Cells

**Figure 1:**
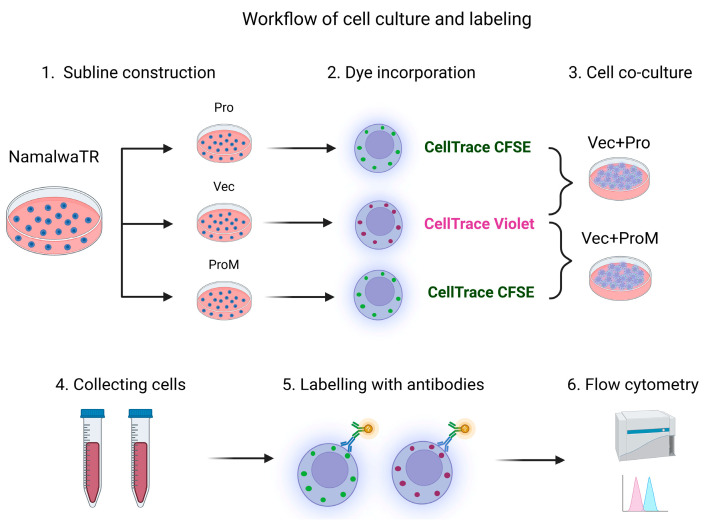
Workflow of cell co-cultures and antibody labeling. The matriptase-positive Namalwa cells (NamaTR-Vec) were labeled with CellTrace Violet (pink letters) and the cells expressing matriptase and prostasin (NamaTR-Pro) or matriptase and an inactive variant prostasin (NamaTR-ProM) were labeled with CellTrace CFSE (green letters). The NamaTR-Vec cells were mixed at the 1:1, 1:2, or 1:3 ratio with increasing portions of the prostasin-positive cells and co-cultured for 24 h. The co-cultured cells were labeled with the M32 matriptase antibody and analyzed in a flow cytometer. Vec: empty vector alone, Pro: wild-type prostasin, ProM: protease-dead variant prostasin. The figure was created in BioRender.com.

We have previously shown that prostasin in exosomes induces activation-dependent matriptase shedding from the Namalwa human non-Hodgkin lymphoma B cells in a cell-exosome co-culture system [37]. Consequently, the proliferation and migration of these cells were reduced. In this study, we established an autologous prostasin–matriptase activation cascade using genetically modified Namalwa cells, as illustrated in Figure 1. The Namalwa sublines of NamaTR-Vec, -Pro, and -ProM were designated for having only the empty vector (Vec), expressing the wild-type human prostasin (Pro), or expressing a protease-dead variant prostasin (ProM). All sublines at 1 × 10^7^ cells/each were labeled with different CellTrace dyes and rested for 24 h in a 37 °C incubator before the co-cultures. The NamaTR-Vec cells (3 × 10^5^), without any prostasin expression and representing the target cells, were co-cultured with the effector cells NamaTR-Pro or NamaTR-ProM at a ratio of 1:1, 1:2, or 1:3, as described in Materials and Methods.

**Figure 2:**
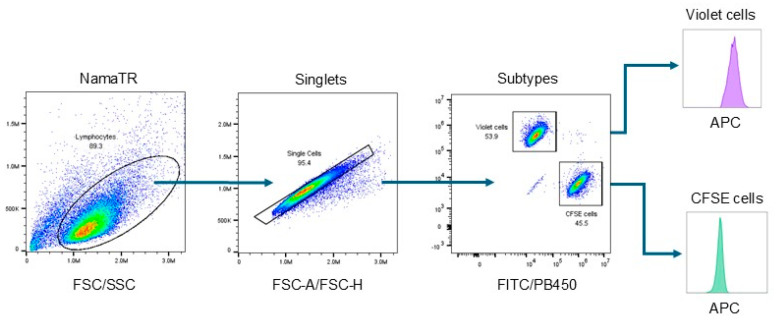
Flow cytometry gating strategy. The Namalwa lymphocytes were gated on the FSC/SSC plot followed by the FSC-A/FSC-H plot for singlets. The subtypes of singlets were further gated on a FITC/PB450 plot for violet-labeled cells and CFSE-labeled cells. For evaluation of matriptase expression, the FITC- or PB450-positive cells were then analyzed separately in the APC channel and presented as a histogram (arbitrary color). In the violet channel (PB450), the histogram represents the matriptase expression in the NamaTR-Vec cells, while that in the CFSE channel (FITC) represents the matriptase expression in the NamaTR-Pro or NamaTR-ProM cells. The figure was created using graphs generated in FlowJo™ v10.9.0 Software (BD Life Sciences, Franklin Lakes, NJ, USA).

After co-culturing and matriptase antibody labeling, the two-colored cells were differentiated in a flow cytometer and the matriptase content in each subtype was analyzed separately, as illustrated in Figure 2. The forward scatter (FSC) versus side scatter (SSC) plot was used to identify live cells. The FSC-A (area) versus FSC-H (height) plot was used to exclude doublets. The FITC versus PB450 plot was used to separate the NamaTR-Vec cells (labeled CellTrace Violet, detected in the PB450 channel) from the NamaTR-Pro or the NamaTR-ProM cells (labeled CellTrace CFSE, detected in the FITC channel). The matriptase expression was then identified in the APC channel (allophycocyanin, conjugated to the secondary antibody) and shown as the histograms.

**Figure 3:**
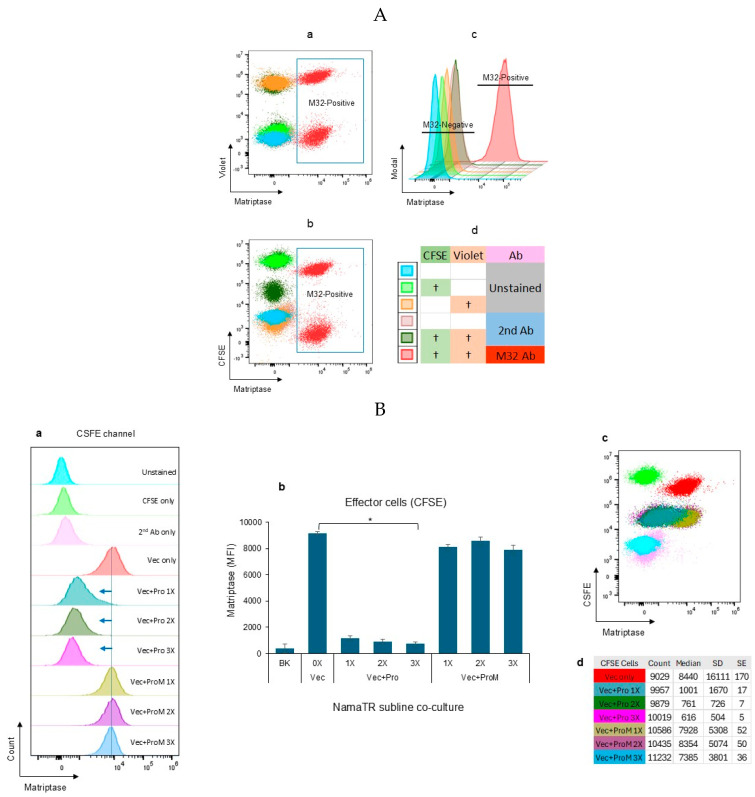

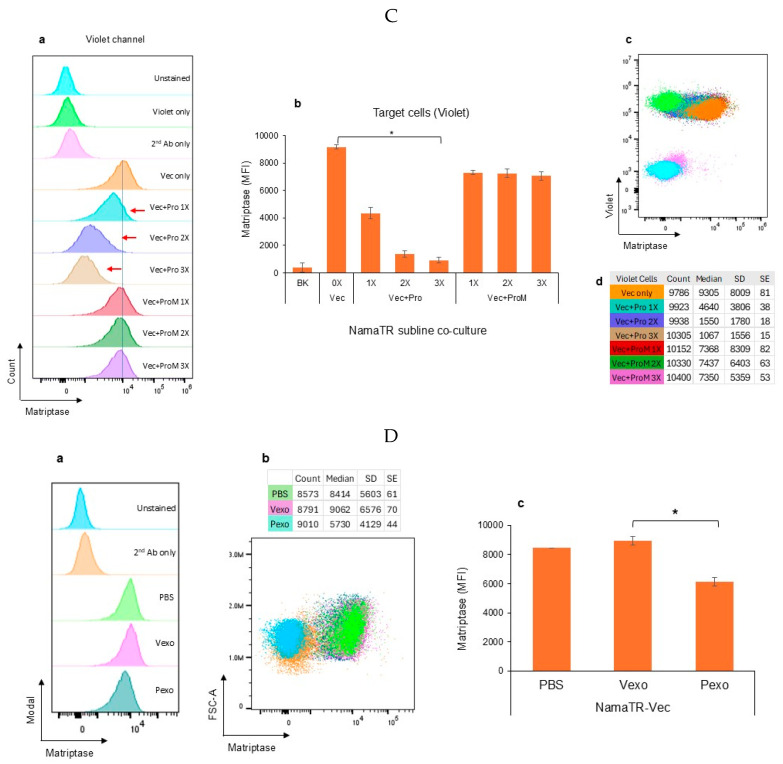
Autologous prostasin–matriptase interaction in the Namalwa cells. (**A**) Flow cytometry plots of the NamaTR cells. a,b: dot plots of control cells and M32-positive cells. c: histograms of all cells. d: coding of staining. “†” indicates the different treatments or conditions of the cells as described in Results. (**B**,**C**) The CFSE NamaTR-Pro, CFSE NamaTR-ProM, Violet NamaTR-Vec cells were co-cultured and stained with the anti-matriptase antibody M32. (**B**)a, (**C**)a: histograms of co-cultured cells. (**B**)b, (**C**)b: bar graphs of matriptase MFI in co-cultured cells. (**B**)c,d, (**C**)c,d: dot plots of cells and the cell count statistics. BK: the background representing the combination of unstained cells, CFSE only (or Violet only), second Ab only; Vec: NamaTR-Vec; Pro: NamaTR-Pro; ProM: NamaTR-ProM; 1×: Vec:Pro cells or Vec:ProM cells at 1:1; 2×: Vec:Pro cells or Vec:ProM cells at 1:2; and 3×: Vec:Pro cells or Vec:ProM cells at 1:3. (**B**,**D**) ANOVA, *p* < 0.05 (*n* = 2). (**D**) Cells were co-cultured with exosomes and stained with the M32 matriptase antibody. a: histograms of NamaTR-Vec-exosome co-cultures. b: dot plots of co-cultured cells and the cell count statistics. c: bar graphs of matriptase MFI in cell-exosome cultures. Vexo: exosomes from cells carrying the empty vector; Pexo: exosomes from cells carrying the prostasin protease. PBS was used as the reagent control. The asterisk (*) denotes *p* < 0.05 (*n* = 3). Representative cell count statistics, including the M32 staining intensity (Median), SD (standard deviation), and SE (standard error), are presented for (**B**–**D**).

The NamaTR cells were characterized by flow cytometry to establish the cell boundaries and the gates for differentiating the matriptase antibody-labeled cell populations (M32-positive). In Figure 3A(a,b), the overlaid dot plots represent the NamaTR cells under different treatments or conditions, including the uncolored and unstained, the uncolored but stained with the secondary antibody alone (2nd Ab), the double-colored (CSFE and Violet) with 2nd Ab staining, the single-colored (CSFE or Violet), and the double-colored (CFSE and Violet) with the M32 matriptase antibody staining. The overlaid histograms (Figure 3A(c)) clearly separated the M32-positive cells from the M32-negative cells. The sample treatments and conditions are coded by Figure 3A(d).

In Figure 3B(a), the NamaTR-Pro cells (Pro: 1×, 2×, 3×) in the CFSE channel were shown to have a trace amount of matriptase remaining, as indicated by the left-shifted peaks (as indicated by the blue arrows) in comparison to the NamaTR-Vec cells. The latter is the target cell without prostasin. The median fluorescence intensity (MFI) was calculated for each sample and shown in the bar graph (Figure 3B(b)). The representative standard errors are shown for the dot plots (Figure 3B(c,d)). About ninety percent of the matriptase protein was removed from these cells. The amount of the matriptase protein in the NamaTR-ProM cells (ProM: 1×, 2×, 3×), however, was maintained at a level comparable to that in the NamaTR-Vec cells. This phenotype is consistent with our previous report that the protease-dead inactive prostasin variant cannot initiate matriptase activation and shedding [37].

The NamaTR-Vec cells in the Violet channel (Figure 3C) had their matriptase contents reduced, whereas these cells do not make any prostasin themselves. This result suggested that the prostasin protein in the NamaTR-Pro cells acted on the matriptase protein in the NamaTR-Vec cells in the co-culture. On the other hand, the matriptase protein content remained high in the NamaTR-Vec cells when the NamaTR-ProM cells were in the co-culture.

The matriptase removal in the NamaTR-Vec cells was dose-dependent (as indicated by the red arrows), as less and less matriptase remained in the NamaTR-Vec cells with the increasing numbers of the NamaTR-Pro cells added in the co-culture (Figure 3C(a)). At a ratio of 1:3 (Vec+Pro 3×), in cell numbers between NamaTR-Vec and NamaTR-Pro, the matriptase content in the NamaTR-Vec cell was reduced to almost the same level as that in the NamaTR-Pro cells (~10%), as shown in the bar graph in Figure 3C(b). The representative standard errors are shown for the dot plots (Figure 3C(c,d)).

To determine how the prostasin from the NamaTR-Pro cells acted on the matriptase in the NamaTR-Vec cells, we added exosomes carrying prostasin (Pexo) in the culture of NamaTR-Vec cells. As expected, the cell-surface matriptase quantity in the NamaTR-Vec cells was reduced, but not when prostasin-null exosomes (Vexo) were used (Figure 3D). This result suggested that the prostasin protein produced in the autologous cells could initiate matriptase activation and shedding via either cell–cell contact or the exosomes. We have thus established that prostasin can induce matriptase shedding when both are co-expressed in the same cells (NamaTR-Pro), but can also do so in an autologous paracrine fashion in the NamaTR-Vec and NamaTR-Pro co-cultures.

### 3.2. Autologous Prostasin–Matriptase Serine Protease Interaction Reduced the Recombinant Matriptase Content in the Jurkat Human Acute Leukemic T Cells

**Figure 4:**
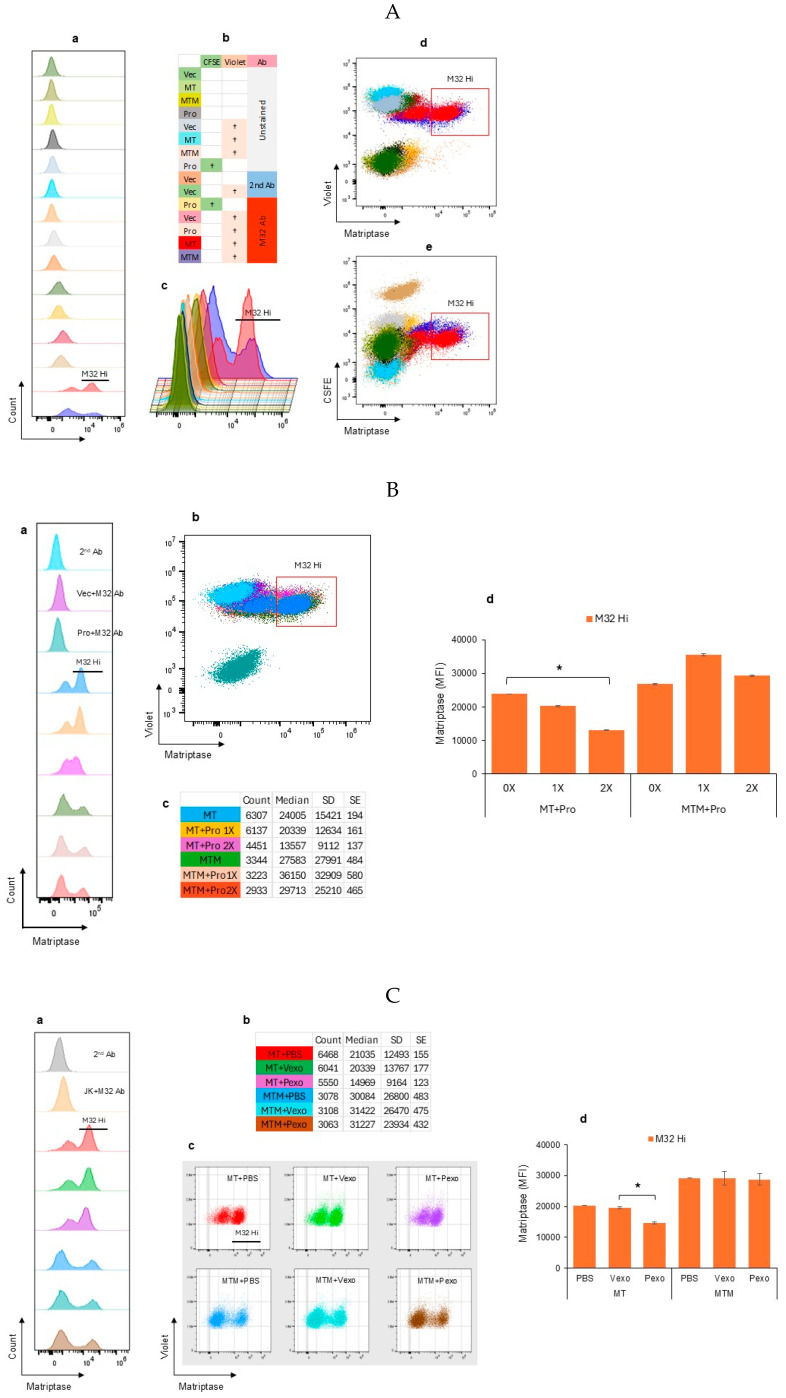
Autologous prostasin–matriptase interaction in the Jurkat cells. (**A**) Flow cytometry plots of the JKTR cells. a,b,c: histograms of control cells and M32-positive cells, and coding of staining. “†” indicates the different treatments or conditions of the cells as described in Results. d,e: dot plots of the cells with matriptase-positive cell populations outlined as M32 Hi. (**B**) Co-cultures of the Jurkat sublines. a–c: flow cytometry plots, cell count statistics. The matriptase-positive cell populations are identified as M32 Hi. The cell counts are the cells of the M32 Hi population. Vec+M32 Ab: JKTR vector cells labeled with the M32 matriptase antibody. Pro+M32 Ab: JKPro cells labeled with the M32 matriptase antibody. d: bar graphs of the median fluorescence intensity (MFI) of matriptase expression in the JKTR-MT (MT) or the JKTR-MTM (MTM) cells in the presence of the JK-Pro (MT+Pro or MTM+Pro) cells at a ratio of 1:0, 1:1, or 1:2. (**C**): a,b,c: flow cytometry plots of the Jurkat cells co-cultured with exosomes and cell count statistics. d: bar graphs of the MFI of matriptase expression. Vexo: exosomes from cells carrying the empty vector, Pexo: exosomes from cells carrying the prostasin protease. PBS was used as the controls without any exosome. (**B**,**C**): the asterisk (*) denotes *p* < 0.05 (*n* = 3). Representative cell count statistics including the M32 staining intensity (Median), SD (standard deviation), and SE (standard error), are presented for (**B**,**C**).

To extend our findings to a broader scope with regard to hematological cells, we selected the Jurkat human acute leukemic T cells as a target for prostasin action. The Jurkat cells do not express either matriptase or prostasin. We genetically engineered Jurkat sublines JKTR-MT to express the wild-type human matriptase, JKTR-MTM to express a protease-dead variant matriptase, and JK-Pro to express the wild-type human prostasin, as described in Materials and Methods. By means of flow cytometry analysis, we show that co-culturing of the JK-Pro cells with the JKTR-MT cells or the JKTR-MTM resulted in a reduction in the matriptase protein quantity in the JKTR-MT cells, but not in the JKTR-MTM cells (Figure 4A).

The Jurkat sublines were characterized by flow cytometry to establish the cell boundaries for populations positive for the M32 matriptase antibody. As shown in Figure 4A, the JK-TR sublines of Vec, MT, MTM, and JK-Pro, uncolored or colored (CFSE or violet), unstained or stained with the secondary antibody only, all had less MFI than that in the MT or MTM cells stained with the M32 matriptase antibody.

The matriptase quantity reduction was enhanced when more JK-Pro cells were added in the co-culture at a ratio of 2:1 (2×) (Figure 4B). Further, when prostasin exosomes (Pexo) were co-cultured with the JKTR-MT or JKTR-MTM cells (Figure 4C), the matriptase quantity was reduced in the JKTR-MT cells, but not in the JKTR-MTM cells. These results indicated that the protease activity of both prostasin and matriptase is required for the cell-surface prostasin–matriptase proteolytic activation cascade and for the activation-induced matriptase shedding.

### 3.3. Prostasin Exosomes Induced Changes in the Cluster Differentiation Molecules (CDs) in Activated Human Peripheral Mononuclear Cells (PBMCs)

**Figure 5:**
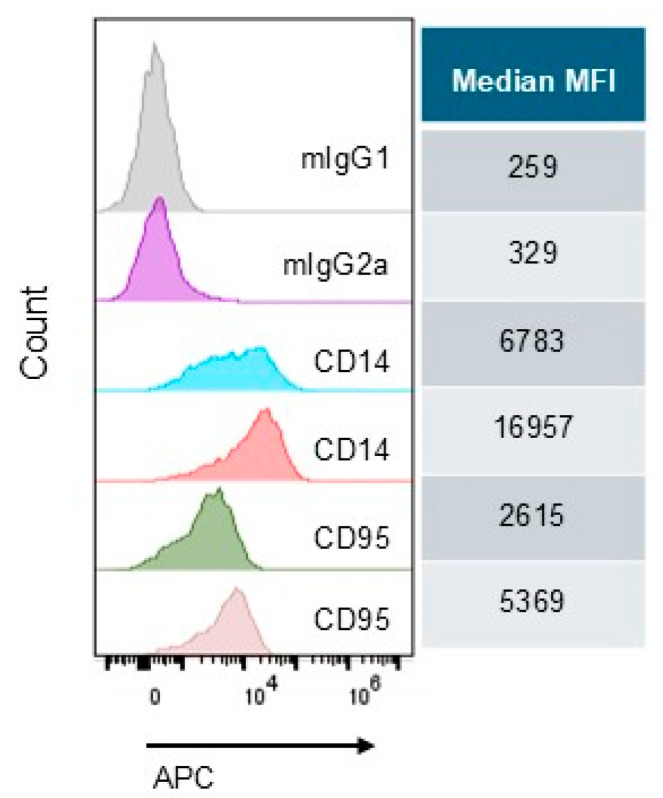
Flow cytometry histograms representing the prostasin exosome-induced CD14 and CD95 expression changes in activated human PBMCs. mIgG1: mouse IgG1 control antibody for CD95, mIgG2a: mouse IgG2a control antibody for CD14. Median fluorescence intensity (MFI) values are presented to the right of each sample. The data were analyzed with the FlowJo™ v10.9.0 Software (BD Life Sciences, Franklin Lakes, NJ, USA).

The effector protease prostasin can be present in the blood in the exosomes, especially in the disease states [2], and the target protease matriptase is present in cancerous B lymphocytes. We set forth to evaluate the impact of prostasin exosomes on the cluster differentiation (CD) molecules in activated human peripheral blood mononuclear cells (PBMCs). The experiment was performed by using the Human Cell Surface Marker Lyoplates kit (BD Biosciences, Franklin Lakes, NJ, USA). A total of 206 antibodies (Table S1) were used in the screening and 174 showed positive staining with an MFI over the control background (unstained, or 2nd antibody only). Among these, 73 showed an increase and 101 showed a reduction, respectively, in the prostasin exosome-treated sample (Pexo) in comparison to the vector exosome-treated sample (Vexo). Overall, the expression of the CDs and surface markers was not significantly changed, except for CD14 and CD95, both of which had an over twofold increase in the MFI in the Pexo-treated samples, as shown in Figure 5.

### 3.4. Gene Expression Profiling (RNA-Seq) of ST14, PRSS8, SPINT1 and SPINT2 (SPSS) Reveals Distinctive Patterns of Expression in Hematological Versus Epithelial Cells

In epithelial cells, matriptase (ST14), prostasin (PRSS8) and their cognate inhibitors HAI-1 (SPINT1) and HAI-2 (SPINT2) are co-expressed and function in an interactive network, the SPSS. Prostasin and matriptase can activate each other to regulate the amounts of each protein present on the cell membrane. The prostasin–matriptase proteolytic activation cascade is tightly regulated by HAI-1 and HAI-2.

We first interrogated the gene expression profiles of *ST14*, *PRSS8*, *SPINT1*, and *SPINT2* (SPSS) in a cohort of eight common carcinomas of epithelial origin in the TCGA Pan-Cancer (PANCAN) dataset using the UCSC Xena Functional Genomics Browser (University of California Santa Cruz, https://xenabrowser.net/, accessed on 20 December 2024). The median log2fpkm (normalized value + 1) of each gene’s expression is 12.5 for *ST14*, 12.0 for *PRSS8*, 12.1 for *SPINT1*, and 13.4 for *SPINT2* (Figure S1), respectively. This narrow range of median values suggested a rather balanced gene expression for the SPSS in epithelial carcinomas.

The SPSS genes are not known to be expressed in hematological (blood) cancer cells; however, B-cell lymphomas were reported to over-express matriptase without the co-expression of its interactive and regulatory proteins of the SPSS network [29,30,31]. This is reflected at the gene expression level, as shown in Figure S2. The *ST14* expression level is high in Burkitt lymphoma (BL, median = 5.8) and diffuse large B-cell lymphoma (DLBC, median = 5.2) but very low in acute myeloid leukemia (LAML, median = 1.5), although all are at much lower levels in comparison to those of epithelial carcinomas (Figure S1). More importantly, there is an imbalanced gene expression profile of SPSS in blood cancer cells. This suggests that matriptase activation and inhibition in B-cell lymphomas may not be regulated in the same way as those in epithelial cells due to the extremely low expression levels of the matriptase regulatory proteins.

**Figure 6:**
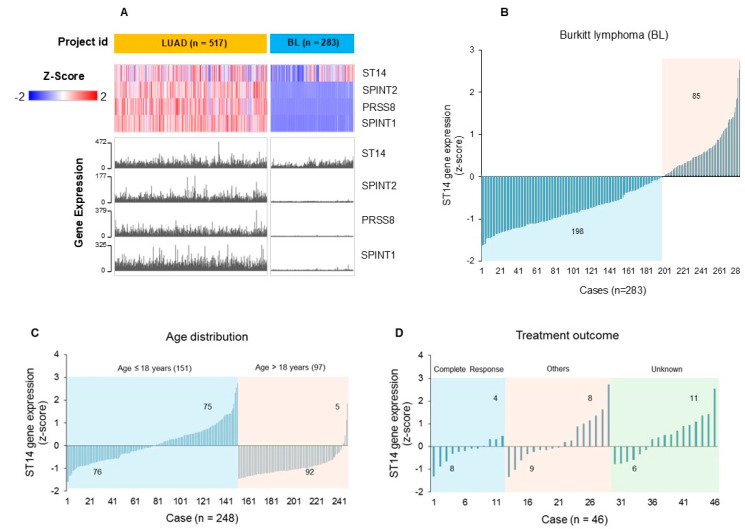
SPSS gene expression in lung adenocarcinoma (LUAD) and Burkitt lymphoma (BL). (**A**) In the heatmap, each row represents a gene, as indicated by the gene symbol (*ST14*, *SPINT2*, *PRSS8*, *SPINT1*). Each column represents a sample, and the number of samples is indicated in the parenthesis next to the project id (LUAD, BL). The level of gene expression is colored blue to red for low to high z-scores. (**B**–**D**): Bar graphs of the *ST14* gene expression levels in BL and in relation to age and treatment outcomes. The numbers above or below the x-axis are the number of cases with positive z-scores or negative z-scores for *ST14* expression, respectively, in total cases, or age distribution or treatment outcome stratifications. Other: combined treatment outcomes of partial response, progressive disease, stable disease, and persistent disease.

We took a closer look at the expression profiles of the SPSS genes in Burkitt lymphoma as this tumor type has been shown to have high levels of matriptase expression [29,30]. We built a BL cohort in the Genomic Data Commons (GDC) Data Portal (https://portal.gdc.cancer.gov/, accessed on 24 December 2024) and visualized the SPSS gene expression heatmap using the LUAD dataset as the reference for matriptase expression levels. As shown in Figure 6A, the SPSS genes are all expressed in the LUAD tumor samples, while in the BL samples *ST14* is expressed at a detectable level in some cases (in the red color), but without much prostasin or HAI co-expression (in the blue color).

In 283 cases, 85 (30%) have a z-score above zero indicating an *ST14* expression level above the mean in this cohort, as shown in Figure 6B. A negative z-score indicates a gene expression level below the mean in the cohort. Further, more cases with positive *ST14* z-scores are seen in children under 18 years of age (75 out of 151 cases, or ~50%), while only 5 out of 97 adult cases over 18 years of age have positive *ST14* z-scores, as shown in Figure 6C. For the treatment outcomes, more cases with lower levels of *ST14* expression (negative z-score) are seen in the complete response group (8 out of 12, or 67%), as shown in Figure 6D. Regarding other treatment outcomes, including partial response, progressive disease, stable disease, persistent disease, the numbers of cases with a positive or negative *ST14* z-score are rather evenly distributed, but the positive *ST14* z-scores are higher (between 0.5 and 3.5) above the mean than those in the complete response group (z-scores < 0.5). Overall, the results indicate that high *ST14* expression levels are found more frequently in tumors of Burkit lymphoma in children and in cases not responding completely to treatments.

## 4. Discussion

The extracellular membrane serine proteases matriptase and prostasin had long been regarded as strictly expressed and functional in the epithelial cells, but recently their relevance in hematological cells has emerged, specifically in B-cell lymphoma [29,30,31]. Currently NHLs are treated with standard chemotherapy using four drugs known as the CHOP (cyclophosphamide, doxorubicin, vincristine, and prednisone), or immunotherapy using monoclonal antibodies targeting B-cell surface proteins, such as rituximab, targeting the CD20 antigen, alone or in combination with standard chemotherapy (R-CHOP) (American Cancer Society). Immunotherapy with chimeric antigen receptor T (CAR-T) cells provides patients with a potential long-term treatment option, especially for patients in disease relapse or who are refractory to conventional therapies such as chemotherapy, radiation, or hematopoietic stem cells (HSC) transplantation.

In some B-cell lymphoma patients, matriptase is ectopically over-expressed in the tumors. While matriptase appears to be an excellent candidate target for treating the NHL, conventional methods to achieve this with antibodies or gene-silencing agents rely on the entry of the agents into the target cells. Previously, we explored prostasin exosomes as a tool for matriptase removal in cancerous B cells, taking advantage of a unique biochemical mechanism of prostasin–matriptase activation–elimination, occurring at the cell surface. Such a strategy showed the potential of long-term effects of the prostasin exosomes after delivery. A proteolytic protease activation cascade is formed between prostasin and matriptase in cancer cells. Upon activation, the activated matriptase molecules shed themselves off the cancer cells into the culture medium. The activated matriptase is toxic to the cells, causing cell death [18,48,49]. In this study, we tested the feasibility of using genetically engineered cells producing prostasin as autologous effector cells to achieve the activation and elimination of matriptase from cancerous B cells. The prostasin exosome, or the prostasin-producing autologous cells may be developed as a potential therapeutic agent for targeting matriptase. This method may be used in combination with other treatment options to improve efficacy, especially for pediatric cancer patients, considering their early developmental stages and the long-term harms and risks of existing therapies, such as chronic illnesses or second cancers in adulthood, as well as reproductive issues. It may also be applicable when a patient’s overall health conditions are not fit for the first-line therapy or a second round of chemotherapy or radiation.

In the explorative CD screening assay (Figure 5), CD14 and CD95 (Fas receptor) were found to have significantly increased levels in activated PBMCs in the presence of prostasin exosomes. The physiological meaning of these changes requires an in vivo investigation. CD14 is a GPI-anchored protein that binds to lipopolysaccharides (LPS), which were used in the PBMC stimulation. It is also an important co-receptor with toll-like receptor 4 (TLR4) in LPS-mediated inflammation. CD95, also known as Fas, is a receptor in programed cell death (apoptosis), a process important in the elimination of aberrant immune cells during an infection or self-over-reactive immune cells. CD14 has been reported to be the determining factor in Fas-mediated apoptosis and inflammation [50], involved critically in Fas receptor internalization on the myeloid cells. This could be the switch that directs the myeloid cells from a pro-inflammation state to a pro-apoptotic state. Consequently, the cytokine release will be reduced in the surrounding environment.

Prostasin is a proteolytic regulator of TLR4 and a suppressor of inflammatory cytokine production in an LPS-induced bladder inflammation [51,52,53,54]. In addition, prostasin inhibits inflammatory cytokine expression initiated by protease-activated receptor 2 (PAR2) in prostate epithelial cells [55], but can also activate PAR2 via matriptase activation [56]. Increased PAR2 expression was observed in the CD11b^+^/CD14^+^ myeloid cells in human allergic contact dermatitis [57]. Thus, our screening of the CDs affected by the prostasin exosomes presents an initial glimpse into an intricate and complex axis of immune and inflammation regulation involving TLR4-CD14-CD95.

The crosstalk between the immune cells and the epithelial cells is the foundation of the innate barrier immunity. Immune cells can modify the epithelial transcriptome to induce rapid protection by memory recall during infection. Conversely, epithelial cells can present antigens to direct the immune cell functions, harnessing barrier immunity [58,59]. Exosomes can travel everywhere in the body, and the exosomes of the epithelial origin may encounter immune cells locally at sites of origin or in other tissues. Prostasin exosomes released into the blood during disease states, such as cardiovascular disease, diabetes, and cancers [2], or released from the epithelial cells into the immediate interstitial environments, are able to interact with TLR4 or PAR2, during immune-epithelial cell–cell interactions as a mechanism of immune modulation.

The current study is limited to using tissue-cultured cell lines in vitro. In addition, PBMC activation was stimulated in part with PHA instead of via CD3/CD28 activation. In future studies, the observations can be corroborated in vivo using preclinical models, e.g., in mice, to validate the hypothesis that the matriptase in the lymphoid cells could be removed by the prostasin-initiated activation and self-activation.

## 5. Conclusions

**Figure 7:**
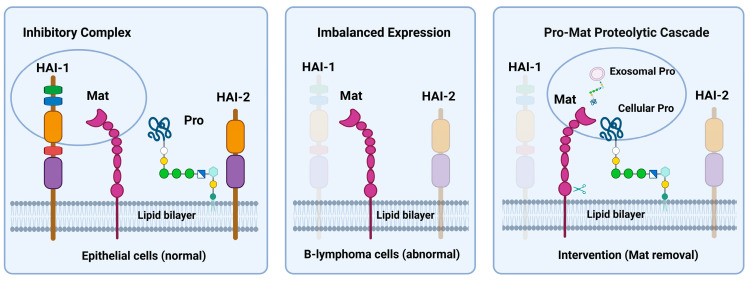
Schematic drawing of the protease–inhibitor network. Matriptase (Mat), prostasin (Pro), HAI-1, and HAI-2 are always co-expressed in normal epithelial cells under physiological conditions. Prostasin can be active while being anchored in the lipid bilayer and regulates matriptase activity via a HAI-2-dependent manner. Active matriptase is hardly seen in the plasma membrane and almost always in a complex with HAI-1. HAI-1 is mostly localized on the cell membrane extracellularly while HAI-2 is predominantly localized intracellularly. In B-cell lymphoma, prostasin is absent, while HAI-1 is either absent or minimally expressed. HAI-2 is at a low level if expressed in these cells. Matriptase, however, is over-expressed, resulting in imbalanced protease–inhibitor expression. The Pro-Mat proteolytic activation cascade initiated by exosomal Pro or cellular Pro can regulate matriptase activity and shedding and may be developed for intervention to remove Mat from the cancerous B cells with matriptase over-expression. The figure was created in BioRender.com.

In this study, we remodeled the prostasin–matriptase proteolytic activation cascade from an exosome-cell mechanism to an autologous cell–cell platform, with a potential application in the intervention of B-cell lymphoma to remove matriptase, as illustrated in Figure 7. B cells migrate from one tissue to another by chemotaxis following an increasing chemokine concentration gradient. Migrating (metastasizing) cancerous B cells with an ectopic matriptase expression may encounter the prostasin protease located in the epithelial cells or in the blood on the exosomes. We provide an initial rationale for prostasin to be incorporated with the CD20- or CD19-CAR-T cells for targeting CD20- or CD19-positive B-cell lymphoma with matriptase over-expression.

## Data Availability

The original contributions presented in this study are included in the article/supplementary material. Further inquiries can be directed to the corresponding authors.

## Supplementary Materials

The following supporting information can be downloaded at https://www.mdpi.com/article/10.3390/cells14040247/s1, Figure S1. Distribution of SPSS expression in a Box and Whisker plot. The dataset contained RNA-seq data from 3959 primary tumors of breast invasive carcinoma (BRCA), colon adenocarcinoma (COAD), esophageal carcinoma (ESCA), lung adenocarcinoma (LUAD) and lung squamous cell carcinoma (LUSC), ovarian serous cystadenocarcinoma (OV), prostate adenocarcinoma (PRAD), and stomach adenocarcinoma (STAD). We established a dataset from 8 common carcinomas in the TCGA database. In this cohort, 3959 primary tumor specimens had been analyzed by RNA-seq. Each box represents the expression distribution of one single gene, i.e., *ST14* or *PRSS8* or *SPINT1* or *SPINT2*, in the 3959 specimens. The graph was generated in Microsoft Excel. Figure S2. Expression of the SPSS genes in epithelial carcinoma and blood cancer cells. The dataset contained carcinoma samples in Figure S1 and the dataset of Burkitt lymphoma (BL, n = 235), diffuse large B-cell lymphoma (DLBC, n = 336), acute myeloid leukemia (LAML, n = 151). The graph was constructed in Microsoft Excel.
