# Supplementary material for: Autologous Paracrine Prostasin–Matriptase Serine Protease Interaction in Lymphoid Cancer Cells
Source: Cells. 2025 Feb 10;14(4):247. doi: 10.3390/cells14040247 (PMC11853515; doi:10.3390/cells14040247)
Supplement: Supplementary file 1 [file cells-14-00247-s001.zip › Tabel S1.pdf]

Table S1. Antibody specificity, clone, and isotype

Specificity	Clone	Isotype	Specificity	Clone	Isotype
CD1a	HI149	Ms IgG 1, κ	CD40	5C3	Ms IgG 1, κ
CD1b	M- T101	Ms IgG 1, κ	CD43	1G10	Ms IgG 1, κ
CD1d	CD1d42	Ms IgG 1, κ	CD44	G44- 26	Ms IgG 2b, κ
CD2	RPA- 2.10	Ms IgG 1, κ	CD45	HI30	Ms IgG 1, κ
CD3	HIT3a	Ms IgG 2a, κ	CD45RA	HI100	Ms IgG 2b, κ
CD4	RPA- T4	Ms IgG 1, κ	CD45RB	MT4	Ms IgG 1, κ
CD4v4	L120	Ms IgG 1, κ	CD45RO	UCHL1	Ms IgG 2a, κ
CD5	L17F12	Ms IgG 2a, κ	CD46	E4.3	Ms IgG 2a, κ
CD6	M- T605	Ms IgG 1, κ	CD47	B6H12	Ms IgG 1, κ
CD7	M- T701	Ms IgG 1, κ	CD48	TU145	Ms IgM, κ
CD8a	SK1	Ms IgG 1, κ	CD49a	SR84	Ms IgG 1, κ
CD8b	2ST8.5H7	Ms IgG2a, κ	CD49b	AK- 7	Ms IgG 1, κ
CD9	M- L13	Ms IgG 1, κ	CD49c	C3 II.1	Ms IgG 1, κ
CD11a	G43- 25B	Ms IgG 2a, κ	CD49d	9F10	Ms IgG 1, κ
CD11b	D12	Ms IgG 2a, κ	CD49e	VC5	Ms IgG 1, κ
CD11c	B- ly6	Ms IgG 1, κ	CD49f	GoH3	Rt IgG 2a, κ
CD13	WM15	Ms IgG 1, κ	CD50	TU41	Ms IgG 2b, κ
CD14	M5E2	Ms IgG 2a, κ	CD51-61	23C6	Ms IgG 1, κ
CD15	HI98	Ms IgM, κ	CD53	HI29	Ms IgG 1, κ
CD15s	CSLEX1	Ms IgM, κ	CD54	LB- 2	Ms IgG 2b, κ
CD16	3G8	Ms IgG 1, κ	CD55	IA10	Ms IgG 2a, κ
CD18	6.7	Ms IgG 1, κ	CD56	B159	Ms IgG1, κ
CD19	HIB19	Ms IgG 1, κ	CD57	NK-1	Ms IgM, κ
CD20	2H7	Ms IgG 2b, κ	CD58	1C3	Ms IgG 2a, κ
CD21	B- ly4	Ms IgG 1, κ	CD59	p282 (H19)	Ms IgG 2a, κ
CD22	HIB22	Ms IgG 1, κ	CD61	VI-PL2	Ms IgG 1, κ
CD23	EBVCS-5	Ms IgG 1, κ	CD62L	Dreg 56	Ms IgG 1, κ
CD24	ML5	Ms IgG 2a, κ	CD63	H5C6	Ms IgG 1, κ
CD25	M- A251	Ms IgG 1, κ	CD64	10.1	Ms IgG 1, κ
CD26	M- A261	Ms IgG 1, κ	CD69	FN50	Ms IgG 1, κ
CD27	M- T271	Ms IgG 1, κ	CD70	Ki-24	Ms IgG 3, κ
CD28	L293	Ms IgG 1, κ	CD72	J4-117	Ms IgG 2b, κ
CD29	HUTS-21	Ms IgG 2a, κ	CD73	AD2	Ms IgG 1, κ
CD30	BerH8	Ms IgG 1, κ	CD74	M-B741	Ms IgG 2a, κ
CD31	WM59	Ms IgG 1, κ	CD75	LN1	Ms IgM, κ
CD32	FL18.26	Ms IgG 2b, κ	CD77	5B5	Ms IgM, κ
CD33	HIM3-4	Ms IgG 1, κ	CD79b	CB3-1	Ms IgG 1, κ
CD35	E11	Ms IgG 1, κ	CD80	L307.4	Ms IgG 1, κ
CD36	CB38 (NL07)	Ms IgM, κ	CD81	JS- 81	Ms IgG 1, κ
CD37	M- B371	Ms IgG 1, κ	CD83	HB15e	Ms IgG 1, κ
CD38	HIT2	Ms IgG 1, κ	CD84	2G7	Ms IgG 1, κ
CD39	TU66	Ms IgG2b, κ	CD85	GHI/75	Ms IgG 2b, κ

Specificity	Clone	Isotype
CD86	2331 (FUN-1)	Ms IgG 1, κ
CD87	VIM5	Ms IgG 1, κ
CD88	D53- 1473	Ms IgG 1, κ
CD89	A59	Ms IgG 1, κ
CD91	A2MR-alpha 2	Ms IgG 1, κ
CDw93	R139	Ms IgG 2b, κ
CD94	HP-3D9	Ms IgG 1, κ
CD95	DX2	Ms IgG 1, κ
CD97	VIM3b	Ms IgG 1, κ
CD98	UM7F8	Ms IgG 1, κ
CD99	TU12	Ms IgG 2a, κ
CD99R	HIT4	Ms IgM, κ
CD100	A8	Ms IgG 1, κ
CD102	CBR-1C2/2.1	Ms IgG 2a, κ
CD103	Ber-ACT8	Ms IgG 1, κ
CD105	266	Ms IgG 1, κ
CD106	51- 10C9	Ms IgG 1, κ
CD107a	H4A3	Ms IgG 1, κ
CD107b	H4B4	Ms IgG 1, κ
CD108	KS-2	Ms IgG 2a, κ
CD109	TEA 2/16	Ms IgG 1, κ
CD112	R2.525	Ms IgG 1, κ
CD114	LMM741	Ms IgG 1, κ
CD116	M5D12	Ms IgM, κ
CD119	GIR- 208	Ms IgG 1, κ
CD120a	MABTNFR1-A1	Ms IgG 1
CD121a	HIL1R- M1	Ms IgG1, κ
CD121b	MNC2	Ms IgG 1, κ
CD122	Mik-beta 3	Ms IgG 1, κ
CD123	9F5	Ms IgG 1, κ
CD124	hIL4R- M57	Ms IgG 1, κ
CD126	M5	Ms IgG1, κ
CD127	hIL-7R-M21	Ms IgG 1, κ
CD128b	6C6	Ms IgG 1, λ
CD130	AM64	Ms IgG 1, κ
CD134	ACT35	Ms IgG 1, κ
CD137	4B4-1	Ms IgG 1, κ
CD137lig	C65- 485	Ms IgG 1, κ
CD138	Mi15	Ms IgG 1, κ
CD141	1A4	Ms IgG 1, κ
CD142	HTF-1	Ms IgG 1, κ
CD146	P1H12	Ms IgG 1, κ

Specificity	Clone	Isotype
CD147	HIM6	Ms IgG 1, κ
CD150	A12	Ms IgG 1, κ
CD152	BNI3	Ms IgG 2a, κ
CD153	D2- 1173	Ms IgG 1, κ
CD154	TRAP1	Ms IgG 1, κ
CD158a	HP- 3E4	Ms IgM, κ
CD158b	CH-L	Ms IgG 2b, κ
CD161	DX12	Ms IgG 1, κ
CD162	KPL-1	Ms IgG 1, κ
CD163	GHI/61	Ms IgG 1, κ
CD164	N6B6	Ms IgG 2a, κ
CD165	SN2	Ms IgG 1, κ
CD166	3A6	Ms IgG 1, κ
CD171	5G3	Ms IgG2 a
CD172b	B4B6	Ms IgG 1, κ
CD178	NOK-1	Ms IgG 1
CD180	G28-8	Ms IgG 1, κ
CD181	5A12	Ms IgG 2b, κ
CD183	1C6/CXCR3	Ms IgG 1, κ
CD184	12G5	Ms IgG 2a, κ
CD193	500000000	Ms IgG 2b, κ
CD195	2D7/CCR5	Ms IgG 2a, κ
CD196	11A9	Ms IgG 1, κ
CD197	2H4	Ms IgM, κ
CD200	MRC OX-104	Ms IgG 1, κ
CD205	MG38	Ms IgG 2b
CD209	DCN46	Ms IgG 2b, κ
CD120b	hTNFR-M1	Rt IgG 2b, κ
CD132	TUGh4	Rt IgG 2b, κ
CD210	3F9	Rt IgG 2a, κ
CD212	2B6/12beta 2	Rt IgG 2a, κ
CD220	3B6/IR	Ms IgG 1, κ
CD221	3B7	Ms IgG 1, κ
CD226	DX11	Ms IgG 1, κ
CD227	HMPV	Ms IgG 1, κ
CD229	HLy9.1.25	Ms IgG 1, κ
CD244	2-69	Ms IgG 2a, κ
CD255	CARL-1	Ms IgG3
CD267	1A1-K21- M22	Rt IgG2a, κ
CD268	11C1	Ms IgG 1, κ
CD271	C40- 1457	Ms IgG 1, κ
CD273	MIH18	Ms IgG 1, κ

**Specificity**	**Clone**	**Isotype**
CD274	MIH1	Ms IgG 1, κ
CD275	2D3/B7-H2	Ms IgG 2b, κ
CD278	DX29	Ms IgG 1
CD279	MIH4	Ms IgG 1, κ
CD282	11G7	Ms IgG 1, κ
CD294	BM16	Rt IgG 2a, κ
CD305	DX26	Ms IgG 1, κ
CD314	1D11	Ms IgG 1, κ
CD321	M.AB.F11	Ms IgG 1, κ
CDw327	E20- 1232	Ms IgG1, κ
CDw328	F023- 420	Ms IgG 1, κ
CD329	E10- 286	Ms IgG1, κ
CD335	9E2/NKp46	Ms IgG 1, κ
CD336	P44- 8.1	Ms IgG1, κ
CD337	P30- 15	Ms IgG1, κ
BLTR-1	P30- 15	Ms IgG1, κ
CLA	HECA-452	Rt IgM, κ
CLIP	CerCLIP	Ms IgG 1, κ
CMRF-44	CMRF44	Ms IgM, κ
CMRF-56	CMRF56	Ms IgG1, κ
GD2	14.G2a	Ms IgG2a
HLA-A2	BB7.2	Ms IgG 2b, κ
HLA-ABC	G46- 2.6	Ms IgG 1, κ
HLA-DQ	TU169	Ms IgG 2a, κ
HLA-DR	G46- 6 (L243)	Ms IgG 2a, κ
HLA-DR,DP,DQ	TU39	Ms IgG 2a, κ
HPC	BB9	Ms IgG1
Integrin β7	FIB504	Rt IgG 2a, κ
MIC A/B	6D4	Ms IgG2a
β2-microglobulin	P44- 8.1	Ms IgG1, κ
fMLP-R	5F1	Ms IgG 1, κ
NKB1	DX9	Ms IgG 1, κ
NK T	6B11	Ms IgG 1, κ
aβTCR	9E2/NKp46	Ms IgG 1, κ
γδTCR	B1	Ms IgG 1, κ
TLR4	TF901	Ms IgG 1, κ
Vβ8	JR2	Ms IgG 2b, κ
Vβ23	AHUT7	Ms IgG 1, κ
