# Supplementary figures and images for: Autologous Paracrine Prostasin–Matriptase Serine Protease Interaction in Lymphoid Cancer Cells
Source: Cells. 2025 Feb 10;14(4):247. doi: 10.3390/cells14040247 (PMC11853515; doi:10.3390/cells14040247)

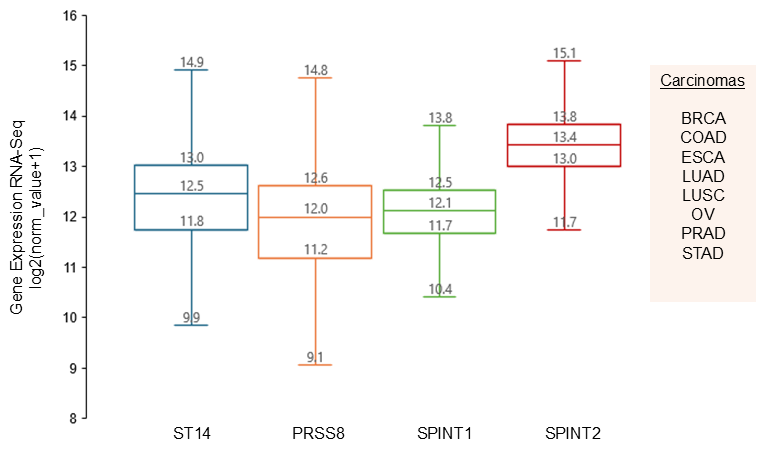

Supplement: Supplementary file 1 [file cells-14-00247-s001.zip › Fig. S1.PNG]

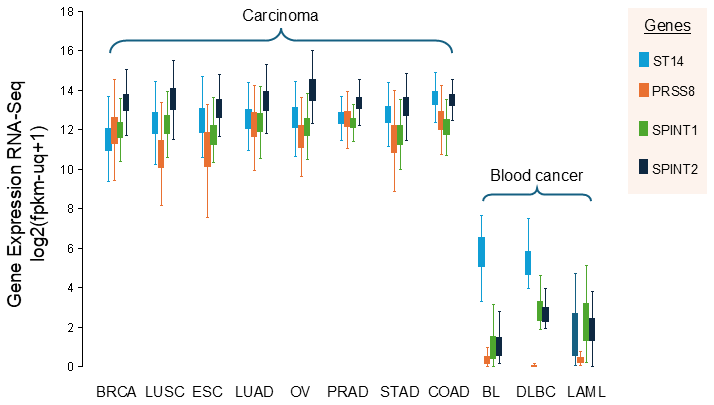

Supplement: Supplementary file 1 [file cells-14-00247-s001.zip › Fig. S2.PNG]
